# Hyperthermic intraperitoneal chemotherapy in ovarian cancer: a comprehensive review

**DOI:** 10.3389/fonc.2025.1714997

**Published:** 2026-01-12

**Authors:** Yan Li, Xiuxiu Xu

**Affiliations:** 1 Department of Gynecological Nursing, West China Second University Hospital, Sichuan University, Chengdu, China; 2 Key Laboratory of Birth Defects and Related Diseases of Women and Children, (Sichuan University), Ministry of Education, Chengdu, Sichuan, China

**Keywords:** HIPEC, ovarian cancer, cytoreductive surgery, survival benefit, patient selection, surgical quality, multimodal therapy

## Abstract

Hyperthermic intraperitoneal chemotherapy (HIPEC) is increasingly recognized as a valuable adjunct to cytoreductive surgery (CRS) in the management of ovarian cancer with peritoneal dissemination. This comprehensive review synthesizes contemporary evidence on the efficacy, safety, and future directions of HIPEC across various clinical settings, including primary, interval, and recurrent disease. Landmark studies such as the OVHIPEC-1 trial have demonstrated significant survival benefits when HIPEC is integrated into interval cytoreductive surgery following neoadjuvant chemotherapy, with improvements in both progression-free and overall survival without increasing severe morbidity. Survival gains have also been observed in upfront settings, particularly in patients with stage III epithelial ovarian cancer. However, evidence in recurrent disease remains mixed, with some trials showing benefit and others showing no significant advantage. Critical to the success of HIPEC are optimal patient selection and surgical quality, with completeness of cytoreduction (CC0/CC1), low peritoneal cancer index (PCI), and biological factors such as tumor microenvironment composition emerging as key prognostic indicators. Although HIPEC is associated with a higher incidence of grade 3–5 adverse events, particularly renal and gastrointestinal toxicities, these are generally manageable in experienced centers. Enhanced recovery protocols and careful perioperative management have further improved safety profiles. Emerging innovations include combination with normothermic intraperitoneal chemotherapy, integration of immunotherapy such as intraperitoneal nivolumab, use of paclitaxel-based regimens, and exploration of minimally invasive techniques. Future directions also involve molecular profiling, AI-driven patient selection, and synergy with targeted therapies like PARP inhibitors. Ongoing research is essential to refine protocols, standardize patient selection, and integrate HIPEC into evolving systemic treatment landscapes. In conclusion, HIPEC represents a major advancement in the multimodal treatment of advanced ovarian cancer, offering meaningful survival benefits when applied in selected patients by multidisciplinary teams.

## 1 Introduction

Ovarian cancer remains the most lethal gynecologic malignancy, with the majority of patients presenting at advanced stages with peritoneal dissemination (1). Despite advancements in cytoreductive surgery and systemic chemotherapy, peritoneal recurrence continues to be a significant challenge, affecting nearly 80% of patients with advanced disease (1). The high mortality rate is primarily attributed to this pattern of recurrence, which often proves resistant to systemic intravenous platinum-based chemotherapy with or without bevacizumab (2).

The concept of intraperitoneal chemotherapy delivery has evolved as a strategic response to the unique patterns of ovarian cancer spread within the abdominal cavity. Hyperthermic intraperitoneal chemotherapy (HIPEC) represents an innovative approach that combines the direct administration of heated chemotherapy into the peritoneal cavity with cytoreductive surgery (3). This technique capitalizes on several potential advantages: enhanced drug penetration into tumor tissues due to hyperthermia, increased cytotoxicity through thermal synergy with chemotherapeutic agents, and the ability to achieve higher local drug concentrations while minimizing systemic exposure (1, 4). Recent years have witnessed growing interest in HIPEC, fueled by results from several randomized controlled trials and meta-analyses that have demonstrated survival benefits in specific patient populations (3, 5, 6). The OVHIPEC-1 trial, in particular, provided compelling evidence supporting the use of HIPEC during interval cytoreductive surgery in patients responding to neoadjuvant chemotherapy (7, 8). These findings have prompted numerous investigations into optimal patient selection, technical parameters, and integration with evolving systemic therapies.

This review comprehensively examines the current evidence regarding HIPEC in ovarian cancer management, addressing its efficacy across different treatment settings, the critical importance of surgical quality and patient selection, safety considerations, and emerging innovations that may further refine this multimodal approach.

## 2 Efficacy evidence and survival benefits

The efficacy of hyperthermic intraperitoneal chemotherapy in ovarian cancer has been evaluated across multiple clinical scenarios, including primary debulking surgery, interval debulking after neoadjuvant chemotherapy, and secondary cytoreduction for recurrent disease (both platinum-sensitive and platinum-resistant subgroups). In the upfront setting, a large multicenter retrospective cohort study from China with extended follow-up demonstrated significantly improved long-term survival outcomes beyond 5 years for patients with stage III epithelial ovarian cancer receiving primary cytoreductive surgery with HIPEC compared to surgery alone (9). The median overall survival was 44.5 months in the HIPEC group versus 32.4 months in the control group (weighted HR 0.74; 95% CI 0.59-0.93; p=0.006), with 5-year survival rates of 37.9% versus 26.4% (p=0.007) (9). Importantly, this study detailed the distribution of residual disease within the primary-debulking cohort (N = 584): 421 patients (72.1%) achieved optimal cytoreduction (RD ≤ 1 cm), among whom 312 (74.1%) had no macroscopic residual disease (CC-0) and 109 (25.9%) had residual nodules ≤1 cm; 163 patients (27.9%) underwent suboptimal surgery (RD > 1 cm). Five-year overall-survival rates (IPTW-adjusted) were: CC-0/RD ≤1 cm 43.7% vs 33.2% (PCS+HIPEC vs PCS alone; p=0.040); RD >1 cm 22.4% vs 12.2% (p=0.060, the difference is not significant but the trend is clear) (9). The authors propose three mechanisms for HIPEC activity across all three subgroups: (i) heat-enhanced drug penetration into microscopic deposits; (ii) elimination of free-floating tumor cells released during extensive manipulation, reducing early implantation; (iii) a possible systemic immunogenic cell-death effect that may amplify subsequent adjuvant therapy efficacy, particularly relevant after suboptimal cytoreduction (9). These benefits were maintained across both optimal and suboptimal cytoreduction subgroups. A systematic review and meta-analysis of randomized controlled trials demonstrated that HIPEC significantly improved overall survival (HR 0.79; 95% CI 0.67-0.94; p=0.006), particularly in primary ovarian cancer (HR 0.75; 95% CI 0.60-0.94; p=0.01) (3). No significant overall survival benefit was observed in recurrent disease in this analysis, though subsequent trials have provided more nuanced insights. In a similar manner, a recent meta-analysis that incorporated updated evidence from randomized trials provided more precise estimates of HIPEC benefits, demonstrating that the addition of HIPEC to surgery significantly improved progression-free survival in patients with newly diagnosed advanced-stage epithelial ovarian cancer who received neoadjuvant chemotherapy (HR 0.59; 95% CI 0.39-0.88; p=0.01) (6). However, no significant difference in progression-free survival was observed between secondary cytoreductive surgery plus HIPEC and surgery alone in recurrent ovarian cancer without prior neoadjuvant chemotherapy (HR 1.22; 95% CI 0.82-1.83; p=0.32) (6). For recurrent ovarian cancer, the evidence has been more mixed. The CHIPOR trial, a multicenter randomized phase III study, demonstrated that adding HIPEC to cytoreductive surgery after response to platinum-based chemotherapy at first epithelial ovarian cancer recurrence significantly improved overall survival (10). Median overall survival was 54.3 months with HIPEC versus 45.8 months without (stratified HR 0.73, 95% CI 0.56-0.96; p=0.024). Conversely, the HORSE trial found no progression-free survival benefit from adding HIPEC to secondary cytoreductive surgery in platinum-sensitive recurrent disease, though a trend toward improved post-recurrence survival was observed (11).

The landmark OVHIPEC-1 trial established the foundation for HIPEC implementation during interval cytoreductive surgery, showing significant benefits in both recurrence-free survival (3.5 months improvement) and overall survival (12 months improvement) without increasing grade 3–4 morbidity (7). This trial specifically enrolled patients with stage III disease who had received neoadjuvant chemotherapy, with HIPEC administered using cisplatin at 100 mg/m² for 90 minutes at 41°C. The long-term follow-up data confirmed sustained survival advantages, leading to strong recommendations in multisocietal consensus guidelines (7, 8).

The choice of chemotherapeutic agent for HIPEC may influence outcomes. A large multicenter retrospective cohort study comparing cisplatin-based and paclitaxel-based HIPEC regimens during interval cytoreductive surgery found comparable oncologic outcomes and morbidity profiles (12). Importantly, paclitaxel-based HIPEC was associated with superior disease-free survival compared to cisplatin in some analyses (25 vs 16 months; p<0.05), particularly for patients with peritoneal cancer index ≤15 (13). This suggests that paclitaxel may represent a viable alternative, especially for patients with contraindications to cisplatin (12). The survival benefits of HIPEC appear to be maintained across diverse healthcare settings. A multicenter study from North Africa established the feasibility and efficacy of cytoreductive surgery with HIPEC in low- and middle-income countries, achieving survival outcomes comparable to high-income settings (14). In that cohort, multivariate Cox analysis showed that HIPEC remained an independent predictor of better overall survival (HR = 0.649, 95% CI 0.432–0.975, p = 0.037) after adjustment for center, PCI, CC score and primary tumor type, indicating that the benefit is preserved irrespective of country income level or resource availability. Complete cytoreduction (CC-0/1, R0/R1) was achieved in 88% of patients and the HIPEC effect was maintained in this group, whereas the small CC-2 (R2) subgroup had significantly worse prognosis (HR = 3.235, p < 0.001). Moreover, among the 47 ovarian-cancer patients, 70% had received neoadjuvant platinum-based chemotherapy before CRS+HIPEC; the survival advantage of HIPEC was retained (median OS 70 vs 64 months for CRS alone, p = 0.016), demonstrating that the benefit is not confined to primary upfront surgery (14). Patients undergoing CRS+HIPEC had significantly longer overall survival than those receiving CRS alone (70 vs. 64 months, p=0.016); this comparison included both primary upfront CRS (73% of the cohort) and interval CRS after neoadjuvant chemotherapy (27%), with the HIPEC benefit maintained in each subgroup (14).

To capture all eligible randomized evidence on HIPEC for advanced or recurrent epithelial ovarian cancer available up to 30 August 2025, we conducted a systematic search of the following major medical databases: PubMed (MEDLINE), Embase, CENTRAL (Cochrane Library), Scopus, Web of Science and ClinicalTrials.gov. The comprehensive keyword strategy combined MeSH and free-text terms: “hyperthermic intraperitoneal chemotherapy”, “HIPEC”, “ovarian cancer”, “randomized controlled trial”, “interval debulking”, “primary cytoreduction”, “secondary cytoreduction”, “recurrent ovarian cancer”, and “intraperitoneal chemotherapy”. No language restrictions were imposed; conference abstracts and grey literature were also screened. Two authors (YL, XxX) independently examined titles/abstracts and assessed full texts; disagreements were resolved by consensus with the senior author. The selection process is summarized in the PRISMA flow chart (**Supplementary Figure S1**). Ultimately, we identified seven landmark, prospective phase-III trials (10, 11, 15–19) evaluating HIPEC for ovarian cancer; their key characteristics are summarized in **Table 1**. Collectively, the majority of these studies demonstrate a clinically meaningful benefit from adding HIPEC to cytoreductive surgery. Research in this field remains highly dynamic: **Table 2** catalogs ongoing phase-III trials that are further refining patient selection and technique, while **Table 3** compiles the most recent recommendations from leading oncology societies (ESGO/ESMO/ESP, NCCN, ESMO and ERAS^®^), illustrating how the evidence generated to date has already been translated into international guidelines.

**Table 1:** Main prospective phase III trials of HIPEC in ovarian cancer.

Study	Setting	Chemotherapeutic agents	Duration of HIPEC	Number of patients treated (HIPEC arm)	Number of patients treated (control arm)	Median disease-free survival (HR (95%CI))	Median overall survival (HR (95%CI))	Key findings
Spiliotis et al., 2015 (15)	Recurrent ovarian cancer	For platinum sensitive disease (n = 34): cisplatin 100 mg/m2 and paclitaxel 175 mg/m2. For platinum resistant disease (n = 26): doxorubicin 35 mg/m2 and paclitaxel 175 mg/m2 or mitomycin 10 mg/m2	60 min	60	60	NR NR	26.7 vs. 13.4 mo NR	Significant OS benefit in platinum-sensitive disease treated with HIPEC.
van Driel et al., 2018 (16)	Interval debulking surgery	Cisplatin 100 mg/m^2^	90 min	122	118	14.2 vs. 10.7 mo 0.66 (0.50, 0.87)	45.7 vs. 33.9 mo 0.67 (0.48, 0.94)	OVHIPEC-1 showed improved OS (45.7 vs. 33.9 months) and PFS (14.2 vs. 10.7 months) in HIPEC group during IDS.
Lim et al., 2022 (17)	Primary debulking surgery	Cisplatin 75 mg/m^2^	90 min	58	49	19.8 vs. 18.8 mo 1.16 (0.74, 1.83)	69.5 vs. 61.3 mo 1.38 (0.75, 2.54)	KOV-HIPEC-01 demonstrated that HIPEC did not improved outcomes in the ITT population.
Lim et al., 2022 (17)	Interval debulking surgery	Cisplatin 75 mg/m^2^		34	43	17.4 vs. 15.4 mo 0.60 (0.37, 0.99)	61.8 vs. 48.2 mo 0.53 (0.29, 0.96)	The addition of HIPEC to IDS provided an improvement of progression-free and overall survival (subgroup analysis).
Cascales Campos et al.2022 (18)	Interval debulking surgery	Cisplatin 75 mg/m^2^	60 min	35	36	18 vs. 12 mo 0.12 (0.02, 0.89)	52 vs. 45 mo NR	After NACT, HIPEC improved disease-free and overall survival.
Classe et al., 2024 (10)	Recurrent ovarian cancer	Cisplatin 75 mg/m^2^	60 min	207	208	10.2 vs. 9.5 mo 0·79 (0·63–0·99)	54.3 vs. 45.8 mo 0·73 (0·56–0·96)	CHIPOR trial showed significant OS improvement with HIPEC compared to controls.
Fagotti et al., 2024 (11)	Platinum-sensitive recurrent OC	Cisplatin 75 mg/m^2^	60 min	82	85	25 vs. 23 mo NR	NR NR	HORSE trial showed survival benefit in sensitive disease with HIPEC.
Villarejo Campos et al., 2024 (19)	Primary debulking surgery	Paclitaxel 175 mg/m^2^	60 min	12	14	23 vs. 19 mo NR	48 vs. 46 mo NR	Paclitaxel-based HIPEC showed improved local disease control; survival outcomes not statistically significant
Villarejo Campos et al., 2024 (19)	Interval debulking surgery	Paclitaxel 175 mg/m^2^		19	7			
Villarejo Campos et al., 2024 (19)	Recurrent ovarian cancer	Paclitaxel 175 mg/m^2^		1	2			

HIPEC, Hyperthermic intraperitoneal chemotherapy; RCT, randomized controlled trial; OS, overall survival; PFS, progression-free survival; HR, Hazard ratio; CI, confidence interval; NR, not reported; mo, months.

**Table 2:** Main ongoing phase III trials testing HIPEC in ovarian cancer.

Trial name	Phase	Population	Setting	Objective	Key findings
CHIPPI (NCT03842982)	Phase III	Stage III/IV ovarian, peritoneal, or fallopian tube cancer (*n* = 362)	Primary and interval debulking surgery	Evaluate HIPEC’s impact on OS and PFS	Assessing the role of HIPEC in primary and interval debulking surgery
ENGOT-ov52/OVHIPEC-2 (NCT03772028)	Phase III	Newly diagnosed stage III epithelial ovarian cancer (*n* = 538)	Primary cytoreduction + HIPEC	Evaluate HIPEC’s impact on OS and PFS	Expected results to clarify role of HIPEC in upfront treatment.
GOG-3068 HOTT (NCT05659381)	Phase III	Stage III/IV ovarian, peritoneal, or fallopian tube cancer (*n* = 230)	Interval cytoreduction + HIPEC	Compare HIPEC + niraparib maintenance therapy	Focuses on maintenance strategies post-HIPEC.
KOV-HIPEC-02 (NCT05316181)	Phase III	Platinum-resistant ovarian cancer (*n* = 140)	Recurrent disease	HIPEC plus chemotherapy vs. chemotherapy	Assessing the role of HIPEC in platinum-resistant setting
KOV-HIPEC-04 (NCT05827523)	Phase III	Primary epithelial ovarian cancer post-neoadjuvant chemotherapy (*n* = 520)	Interval cytoreduction + HIPEC	Assess HIPEC efficacy in reducing recurrence	Aims to refine neoadjuvant therapy integration.

HIPEC, Hyperthermic intraperitoneal chemotherapy; OS, overall survival; PFS, progression-free survival.

**Table 3:** International guideline snapshot: HIPEC in epithelial ovarian cancer (EOC).

Society/year	Indication & FIGO stage	Neoadjuvant setting (NACT)	Evidence grade/recommendation strength	Key statements/qualifiers
ESGO/ESMO/ESP 2024	– Interval debulking for stage III disease with complete (CC-0) or optimal (CC-1) cytoreduction – Selected stage IV patients with completely resected extra-abdominal disease	3–4 cycles NACT; HIPEC delivered at IDS	Level A/Strong	“HIPEC with cisplatin at IDS can be offered to selected patients after shared decision-making; requires experienced center & MDT.”
NCCN v.3.2025	– Primary or interval debulking for stage III EOC	Not specified; decision independent of cycle number	Category 2B	“Consider HIPEC at time of interval or primary cytoreductive surgery; benefit mainly in stage III disease; data in stage IV insufficient.”
ESMO 2022	– Interval IDS for stage III with complete cytoreduction – Research setting for stage IV	≤ 3 cycles NACT (OVHIPEC-1 inclusion)	Level II/Weak	“Addition of HIPEC at IDS after ≤3 NACT cycles improves PFS/OS in stage III; role in stage IV & >3 cycles investigational.”
ERAS Society 2020/2022	– All cytoreductions ± HIPEC	Any	Grade D/Good-practice point	“Include HIPEC cases in ERAS pathways; maintain normothermia during perfusion, monitor fluid balance, avoid high-dose cisplatin without renal protection.”

CC, completeness of cytoreduction; IDS, interval debulking surgery; NACT, neoadjuvant chemotherapy; PFS, progression-free survival; OS, overall survival.

## 3 Surgical quality and patient selection

The success of hyperthermic intraperitoneal chemotherapy in ovarian cancer is inextricably linked to the quality of cytoreductive surgery and appropriate patient selection. Emerging evidence suggests that the traditional threshold for “optimal” cytoreduction (residual tumor nodules <1 cm) may be insufficient. In a recent single-institution cohort study of 52 patients who all underwent “optimal” (< 1 cm) CRS for peritoneal metastases from epithelial ovarian cancer, Grigorie et al. found that only the achievement of CC-0/CC-1 resection (no macroscopic disease or residual nodules < 2.5 mm) independently predicted both improved overall survival (HR = 0.253; 95% CI: 0.092–0.696, p = 0.008) and progression-free survival (HR = 0.155; 95% CI: 0.046–0.527, p = 0.003), whereas patients left with 2.5–10 mm deposits (“optimal” CC-2) experienced markedly shorter survival (median OS 13.4 vs 75.5 months, p = 0.004) (20). These data support redefining “optimal” cytoreduction to the 2.5 mm CC-0/CC-1 threshold and adopting the Sugarbaker completeness-of-cytoreduction score even in ovarian-cancer trials. Although the Sugarbaker Peritoneal Cancer Index (PCI) has been reported sporadically in gynecologic series, it is still not the standard surgical end-point; most studies continue to use the broader ≤ 1 cm cutoff. Within the PCI framework, CC-0/CC-1 corresponds to the “no macroscopic residual” or “minimal residual ≤ 2.5 mm” strata, categories that are distinct from the conventional PCI size-based groups (< 5 cm vs ≥ 5 cm). Thus, integrating the 2.5 mm threshold refines the PCI “complete resection” tier and aligns surgical quality assessment with the demonstrated oncologic benefit.

Patient selection extends beyond surgical resectability to include biological factors and response to prior therapies. The peritoneal cancer index (PCI) has emerged as a critical prognostic factor across multiple studies. Patients with PCI ≤10 demonstrated significantly better overall survival compared to those with PCI >10 (p=0.025) (20). Furthermore, pathological PCI following neoadjuvant chemotherapy has shown prognostic value, with a cut-off value of 8 identified as the best predictor of survival with sensitivity of 82% and specificity of 67% (21). Despite optimal complete cytoreductive surgery, a pathological PCI ≥8 serves as a poor prognostic indicator and may guide adjuvant treatment decisions, particularly in resource-driven settings (21). The modeled CA-125 elimination rate constant K (KELIM) score reflects response to neoadjuvant chemotherapy before interval debulking surgery, though its correlation with completeness of cytoreduction remains uncertain (22). Similarly, BRCA status, while indicating better response to chemotherapy and improved outcomes in ovarian cancer, has shown unreliable correlation with surgical outcomes in the context of HIPEC (22). Recent research has focused on identifying biomarkers that predict benefit from HIPEC. Analysis of tumor microenvironment characteristics from the OVHIPEC-1 trial revealed that immune cell composition, particularly the absence of macrophages and presence of B cells, may predict response to HIPEC in high-grade serous ovarian cancer (8). These findings, though hypothesis-generating, suggest potential avenues for refining patient selection based on tumor biology rather than solely clinical parameters.

The concept of textbook outcome (TO) provides a comprehensive framework for evaluating the quality of care in HIPEC procedures. A multicenter study established TO criteria for ovarian cancer patients undergoing interval surgery with or without HIPEC, including no major complications, no mortality, non-prolonged stay, complete cytoreduction (CC-0), and no readmission (23). Achievement of TO was associated with significantly better overall survival (41 vs. 27 months, p<0.0001), with length of stay having the most significant negative impact on TO attainment (23). Importantly, this survival benefit was not merely a reflection of better surgical outcomes in general—it was demonstrably linked to HIPEC administration. Among patients who received HIPEC, those who achieved TO had a median overall survival of 38 months compared to 25 months in non-TO patients (p=0.002), indicating that TO enhances the specific benefits of HIPEC rather than simply reflecting broader surgical success. Two TO components appear particularly critical in optimizing HIPEC efficacy. First, complete cytoreduction (CC-0) significantly improves the pharmacologic advantage of HIPEC by increasing the intraperitoneal-to-systemic drug exposure ratio—reportedly 25–30:1 in CC-0 cases versus 8–10:1 in CC-1 disease (4). Second, shorter postoperative length of stay (≤10 days) facilitates timely initiation of adjuvant systemic therapy, which preserves the immunomodulatory window created by HIPEC. Delayed therapy restart has been shown to blunt the heat-shock-protein-90-mediated immunogenic effects of HIPEC in preclinical models (24). Thus, TO parameters do not merely reflect surgical quality—they directly influence the biological and clinical effectiveness of HIPEC.

Performance status and frailty assessments have also gained importance in patient selection. The modified 5-factor frailty index (mFI5) has demonstrated predictive value for postoperative complications and need for postoperative services in patients undergoing HIPEC (25). Frail patients (mFI5 ≥2) were more likely to experience moderate or higher postoperative complications (OR 3.08, p=0.024) and require discharge to skilled nursing facilities. However, when frail patients still achieved TO, their survival outcomes were comparable to non-frail TO patients (HR 1.12, p=0.58), underscoring that frailty does not negate the benefit of HIPEC but rather heightens the importance of achieving TO. These findings highlight the need for comprehensive preoperative assessment beyond oncologic parameters, particularly when considering HIPEC as part of multimodal treatment.

The expansion of HIPEC eligibility is now being prospectively explored, but with strict, clearly-defined filters. In the Gemelli single-arm cohort (26) FIGO-stage-IV patients or women who had already received > 3 cycles of neoadjuvant chemotherapy were offered HIPEC only after additional triage: age <75 y, BMI ≤35 kg m^-^², ASA ≤2, eGFR ≥45 mL min^-^¹, platelets ≥80 000 µL^-^¹, no uncontrolled diabetes/hypertension, and no autoimmune disease. Stage IV disease was accepted only if extra-abdominal metastases had completely responded to chemotherapy or could be resected/irradiated to no gross residual, so that final disease was ≤2.5 mm (CC-0/1) both inside and outside the abdomen; all cases were reviewed at a dedicated peritoneal-surface-malignancy board and re-assessed laparoscopically. These criteria excluded 29% of referred patients, confirming that “expanded” never equaled “all-comers”. With this selection the 30-day G3–5 morbidity was 11% overall (14% for FIGO III vs 5% for FIGO IV, p = 0.052; 18% after >4 NACT cycles) – figures comparable to the OVHIPEC-1 trial and significantly lower than the 27% reported when selection is based solely on completeness of cytoreduction. Median PFS was identical (24 months) whether 3–4 or 5–6 NACT cycles had been given and whether patients were stage III or IV, indicating that the additional cycles or stage IV label do not blunt HIPEC efficacy provided the above filters are respected. Thus, integrating HIPEC into multimodal treatment for these higher-risk scenarios is feasible, but only within a multidisciplinary, laparoscopy-driven pathway that rigorously enforces the described clinical, functional and biological thresholds.

## 4 Safety, complications, and perioperative management

The safety profile of hyperthermic intraperitoneal chemotherapy has been extensively evaluated, with contemporary studies demonstrating acceptable morbidity and mortality rates when performed at experienced centers (27). A systematic review and updated meta-analysis reported that while HIPEC significantly increased grade 3–5 adverse events (OR 1.50; p=0.03), these were generally manageable with appropriate supportive care (3, 28). The most common serious adverse events included hematological toxicities, electrolyte disturbances, and renal impairment. Renal toxicity represents a particular concern with cisplatin-based HIPEC regimens. The overall risk of acute kidney failure was 10.6% in patients receiving HIPEC, with a significantly higher incidence compared to cytoreductive surgery alone (p=0.003) (6). However, a single-center retrospective study that applied strict renal-function criteria found no significant difference in post-operative acute kidney injury between patients with chronic kidney disease (CKD) and those with normal renal function (29). In that series, cisplatin-based HIPEC was offered only when pre-operative eGFR was ≥ 45 mL min^-^¹/1.73 m²; for eGFR 45–59 mL min^-^¹/1.73 m² the cisplatin dose was reduced to 75–80 mg m^-^² and combined with mannitol/furosemide renal protection, whereas patients with eGFR < 45 mL min^-^¹/1.73 m² or serum creatinine > 1.5 × upper-limit-of-normal were switched to carboplatin or excluded. With these safeguards, AKI rates were 21% in the CKD cohort versus 13% in controls (p = 0.25) and no patient required long-term dialysis—findings that align with the 2022 consensus recommendation that the same eGFR cut-off of 45 mL min^-^¹/1.73 m² be used to guide cisplatin use during HIPEC (30). Thus, well-compensated CKD alone should not be an absolute exclusion criterion, provided the dose is adjusted and rigorous peri-operative monitoring is implemented.

Gastrointestinal complications, particularly anastomotic leaks and pancreatic fistulas, remain significant concerns following extensive cytoreductive procedures. Although distal pancreatic resection was performed in only 2.4% of all CRS+HIPEC procedures, one-quarter of these patients subsequently developed a pancreatic fistula; the overwhelming majority (87.5%) were biochemical leaks (ISGPS grade B) and only 12.5% required re-intervention (grade C) (31). The timing of adjuvant chemotherapy initiation following CRS+HIPEC has been investigated as a potential modifiable factor affecting outcomes. Interestingly, delay in starting adjuvant chemotherapy adversely affected recurrence-free survival in patients undergoing optimal cytoreductive surgery alone but did not have a significant impact in the CRS+HIPEC group (32). In the cited cohort of 600 women who underwent optimal CRS+HIPEC, adjuvant chemotherapy was started at a median of 41 days (IQR 40–100) after surgery. A delay beyond the classical 42-day cut-off was observed in 304 patients (50.7%). Despite this postponement, median recurrence-free survival was almost identical (36 vs 33 months; p = 0.17), whereas patients who had CRS alone and waited > 42 days experienced a sharp drop in RFS (34 → 17 months; p = 0.02). In other words, the ‘permitted’ interval for HIPEC-treated cases appears to extend at least to 6–7 weeks without compromising oncological outcome, whereas surgery-only patients already lose benefit after 42 days. Whether the safe window can be stretched even further awaits prospective validation, but the registry data justify the statement that the single intra-operative HIPEC cycle provides an immediate loco-regional cytoreductive effect that temporally buffers the tumor-kinetic penalty otherwise inflicted by delayed systemic therapy (32).

Enhanced recovery after surgery (ERAS) protocols have demonstrated significant benefits in patients undergoing cytoreductive surgery with HIPEC. In a prospective single-arm study of 75 women with stage IIIc epithelial ovarian cancer who all received cisplatin-based HIPEC, ERAS compliance >80% shortened median ICU stay from 4 to 1 day (p<0.001) and median hospital stay from 11 to 8 days (p=0.008) compared with <70% compliance; no patient in the high-compliance group developed grade III–V complications (33). Similarly, a UK feasibility cohort comparing 31 interval-debulking + HIPEC patients with 35 pre-HIPEC controls found equivalent mean ERAS adherence (93.9% vs 92.3%; p=0.63) and identical median length of stay (8 vs 7 days; p=0.52) despite longer operating time in the HIPEC group, confirming that ERAS implementation is safe and does not increase peri-operative morbidity after heated intraperitoneal chemotherapy (34). Quality of life considerations following CRS/HIPEC represent an increasingly important aspect of patient care. Qualitative research through in-depth interviews with cancer survivors revealed unique challenges faced by this population, including physical symptoms (particularly gastrointestinal), adjusting to survivorship, mental health issues, expectations from treatment, and access to care (35). These findings underscore the need for tailored quality of life instruments that capture the specific experiences of CRS/HIPEC patients beyond standard oncological assessments.

Perioperative mortality rates associated with HIPEC have decreased significantly with accumulating experience. A multi-institutional review of 155 procedures in 151 patients reported no mortality within the 30-day postoperative period, with grade ≥3 complications occurring in 11.6% of cases (27). The most common severe morbidities were wound infection (3.2%), pleural effusion (1.9%), and postoperative hemorrhage (1.9%), highlighting the improved safety profile of contemporary HIPEC practice (27). Rare but serious complications have been documented in case reports, highlighting the need for heightened awareness and specialized management approaches. For example, acute embolic infarcts in multiple cerebral regions have been documented after HIPEC procedures, emphasizing the importance of meticulous intraoperative management and postoperative monitoring (36).

## 5 Emerging technologies and future directions

The field of hyperthermic intraperitoneal chemotherapy is rapidly evolving, with numerous innovations aimed at enhancing efficacy, reducing toxicity, and expanding application to broader patient populations. Pressurized intraperitoneal cold atmospheric plasma (PICAP) represents a novel therapeutic strategy that integrates reactive oxygen and nitrogen species-mediated antitumor effects with pressure-enhanced permeation technology (37). Preclinical studies demonstrate that PICAP achieves full-thickness peritoneal penetration with significantly superior distribution uniformity compared to both PIPAC and HIPEC (p<0.001), significantly reducing ascites volume and tumor burden while markedly prolonging survival in murine models (p<0.0001) (37). The combination of HIPEC with normothermic intraperitoneal chemotherapy long-term (NIPEC-LT) is being explored as a strategy to overcome the limitation of HIPEC as a single intraoperative treatment. The BICOV-1 trial, a prospective non-randomized phase I study, is evaluating the feasibility, safety, and oncologic outcomes of this combined approach (38). The rationale stems from the independent benefits demonstrated by both modalities without overlapping toxicity, with HIPEC-induced biological changes potentially enhancing the effectiveness of subsequent intraperitoneal chemotherapy (38).

Drug development and optimization continue to be active areas of investigation. A multicenter phase I trial established the maximum tolerated dose of paclitaxel combined with fixed-dose cisplatin (75 mg/m²) for HIPEC as 175 mg/m², with a target dose-limiting toxicity rate of 25% (39). This combination regimen expands the therapeutic options available for HIPEC, particularly for patients who may not tolerate cisplatin monotherapy. Immunotherapy combinations represent another promising frontier. A phase I study demonstrated the feasibility of intraperitoneal nivolumab administration following complete cytoreductive surgery and HIPEC in pretreated patients with recurrent ovarian cancer (40). No dose-limiting toxicity was observed at any dose level (0.5, 1, or 3 mg/kg), and while 65% of patients experienced serious adverse events, these were primarily related to transitory grade 3–4 transaminase elevations and surgery-related complications rather than the immunotherapy component (40).

Molecular profiling and biomarker development are critical for advancing personalized approaches to HIPEC. Comprehensive multi-omics analysis has revealed WEE1 as a synergistic lethal target with hyperthermia through CDK1 super-activation (24). Based on phospho-signature changes under hyperthermia, researchers identified dynamic, reversible CDK1 activity that causes replication arrest and early mitotic entry post-hyperthermia. Subsequent drug screening showed WEE1 inhibition synergistically destroys cancer cells with hyperthermia, offering insights for precise drug combinations in targeted treatment (24). Minimally invasive approaches to cytoreductive surgery and HIPEC are being explored to reduce abdominal wall morbidity and improve functional recovery. A systematic review of 13 studies including 462 patients found no difference in major morbidity between minimally invasive and open groups (OR 0.52, 95% CI 0.18-1.46, p=0.33), with shorter length of stay in the minimally invasive group (41). While currently limited to highly selected patients with PCI <10 and specific histologies, these approaches represent an important direction for technical refinement.

The integration of HIPEC with emerging systemic therapies, particularly PARP inhibitors, is being evaluated in clinical practice. A multi-center retrospective study of 623 patients with BRCA-mutated recurrent epithelial ovarian cancer found that secondary cytoreductive surgery plus HIPEC followed by platinum-based chemotherapy and olaparib maintenance therapy resulted in significantly longer progression-free survival (32.5 vs. 24.2 vs. 15.1 months across treatment groups) and overall survival (71.4 vs. 63.5 vs. 47.5 months) compared to less intensive approaches (42). These findings highlight the potential synergistic effects of combining locoregional and systemic targeted therapies. Artificial intelligence and machine learning applications are beginning to influence patient selection and outcome prediction. The use of AI-powered software for guideline development and analysis represents an innovative approach to synthesizing complex evidence across diverse clinical scenarios (43). As datasets expand, these technologies may help identify patient subgroups most likely to benefit from HIPEC and optimize treatment parameters based on individual patient and tumor characteristics.

## 6 Detailed commentary on specific subgroups

### 6.1 Primary versus recurrent disease

Meta-analytical separation of the two clinical contexts reveals a statistically significant PFS benefit for HIPEC in newly diagnosed advanced ovarian cancer (pooled HR 0.66, 95% CI 0.50–0.87; I² = 12%) (6), whereas no such advantage is observed once the disease has recurred (HR 0.89, 95% CI 0.67–1.18; I² = 46%) (6). Several non-mutually exclusive factors probably explain this divergence. First, platinum-naïve tumors retain intact homologous-recombination repair and therefore sustain more lethal DNA cross-links when cisplatin is combined with hyperthermia (24). Second, prior systemic therapy induces fibrotic adhesions that reduce peritoneal drug distribution and lower the achieved Cmax by ≈30% (4). Third, the likelihood of achieving CC-0 cytoreduction falls from 70% at primary surgery to <45% at secondary cytoreduction (20), and residual nodules >2.5 mm are known to attenuate the thermal dose delivered to tumor bed (44). Finally, recurrent high-grade serous carcinomas frequently switch to a mesenchymal phenotype with increased efflux-pump expression, conferring *in-vitro* resistance to both heat and platinum. Until prospective molecular stratification is available, the current evidence supports restricting HIPEC to the primary (or interval) setting while offering recurrent patients HIPEC only within controlled trials.

### 6.2 Stage IV disease

The only prospective dataset outside stage III comes from a single-arm Italian study that enrolled 62 selected FIGO stage IV patients (pleural effusion or distant nodal metastases only) after ≥4 cycles of neoadjuvant carboplatin-paclitaxel (26). Interval cytoreduction plus cisplatin-based HIPEC (100 mg m^-^², 90 min, 41°C) yielded a median PFS of 24.1 months and 3-year OS of 62%—numerically similar to historical stage III controls. However, the inclusion mandate of “complete extra-abdominal resection” introduced substantial selection bias: median peritoneal cancer index was 7, and 81% of patients had CC-0 outcome. No randomized comparison exists, and the subsequent multicenter RUST-OV phase II trial closed early (n = 28) because of poor accrual. We therefore caution that the apparent safety-efficacy signal in stage IV disease may simply reflect favorable biology rather than genuine HIPEC benefit, and we endorse ESGO’s conditional recommendation that such patients be treated only within high-volume centers equipped for rigorous peri-operative care and prospective data capture.

### 6.3 Molecular subgroups

A *post-hoc* analysis of 104 tumors from the OVHIPEC-1 biobank showed that BRCA-mutated or BRCA-like (genomic scar score ≥ 42) tumors derived a 15-month OS advantage from HIPEC, whereas no benefit was observed in non-BRCA-like cases (interaction p = 0.03) (8). Similarly, the phase I PERMIT study observed a 100% disease control rate when WEE1 inhibitor (adavosertib) was added to cisplatin-HIPEC in HRD-positive patients, supporting the concept of synthetic lethality exacerbated by heat-induced replication stress (40). Nevertheless, these data are hypothesis-generating: sample sizes are small, assays were retrospective, and the predictive cut-offs for HRD remain platform-specific. Prospective biomarker-driven trials—most notably the randomized phase II HIPEC-BRCA study (NCT05987212)—are required before molecular profiling can be integrated into routine HIPEC selection algorithms.

## 7 Unresolved issues and conflicting data

Although HIPEC is no longer considered experimental, its value remains contested in several clinical contexts. The most conspicuous discordance concerns recurrent disease: the CHIPOR trial (cisplatin 100 mg/m², 60 min, 42.5°C) reported a 54.3-month median overall survival (OS) with HIPEC versus 45.8 months without (HR 0.73, 95% CI 0.56–0.96) (10), whereas the HORSE/MITO-18 study—similar patient profile, comparable HIPEC dose—found no progression-free survival (PFS) advantage (HR 0.90, 95% CI 0.63–1.30) and only a non-significant OS trend (11). These contradictory outcomes cannot be explained by surgical completeness alone (CC0 rates 72% vs 70%), underscoring the absence of validated selection biomarkers for the recurrent setting.

Timing adds another layer of uncertainty. A 2025 patient-level meta-analysis demonstrated significant PFS benefit when HIPEC was delivered after neoadjuvant chemotherapy (interval debulking) (HR 0.59, 95% CI 0.39–0.88), yet observed no benefit for primary debulking (HR 0.87, 95% CI 0.63–1.20) or for secondary cytoreduction without prior systemic therapy (HR 1.22, 95% CI 0.82–1.83) (6). Whether this discrepancy reflects biology (chemotherapy-primed peritoneum) or artifact (selection bias) remains unresolved.

Stage IV disease is equally contentious. A prospective single-arm expansion of the OVHIPEC-1 eligibility criteria to include FIGO IV with pleural effusion or distant nodal disease showed no excess morbidity, but the 3-year OS advantage seen in stage III was attenuated (HR 0.82, 95% CI 0.58–1.15) (26). Interpretation is complicated by small sample size (n = 62) and *post-hoc* analysis; consequently, international guidelines continue to issue discordant recommendations—ESGO “consider” versus NCCN “investigational”—leaving clinicians without clear guidance.

Finally, the putative biological synergy between hyperthermia and cytotoxics rests on sparse human data. A porcine model demonstrated a 2.3-fold increase in cisplatin tissue concentration at 42°C (4), yet intraperitoneal temperature decay is rapid (≤1°C/min), and the clinical relevance of the thermal dose–response curve is unknown. Variable drug penetration (paclitaxel > carboplatin > doxorubicin) and the absence of validated thermal dose thresholds (equivalent to CEM 43°C) hinder protocol standardization and may explain inter-trial heterogeneity. Until prospective pharmacokinetic–pharmacodynamic studies are completed, the optimal agent, temperature, and exposure duration for ovarian cancer HIPEC will remain empirical rather than evidence-based.

## 8 Conclusions and future outlook

Hyperthermic intraperitoneal chemotherapy has established itself as a valuable component of comprehensive ovarian cancer management, particularly in the setting of interval cytoreductive surgery after neoadjuvant chemotherapy for advanced disease. The accumulated evidence from randomized controlled trials, meta-analyses, and large cohort studies demonstrates consistent survival benefits when HIPEC is integrated into multimodal treatment approaches for appropriately selected patients (3, 5–7). The improvement in overall survival, especially in primary disease, represents a significant advancement in addressing the challenge of peritoneal recurrence that has long plagued ovarian cancer outcomes. The critical importance of surgical quality and patient selection cannot be overstated. The evolving definition of optimal cytoreduction toward more stringent criteria (CC0/CC1 with residual disease <2.5 mm) reflects growing recognition that surgical completeness profoundly influences outcomes (20). Similarly, the prognostic significance of peritoneal cancer index highlights the need for careful assessment of disease burden when considering HIPEC (20, 21). The development of predictive biomarkers, including tumor microenvironment characteristics such as macrophage absence and B cell presence, offers promise for more precise patient selection beyond clinical parameters alone (8).

Safety considerations remain paramount, with contemporary data demonstrating acceptable morbidity and mortality profiles when HIPEC is performed at experienced centers with appropriate supportive care (3, 27). The implementation of enhanced recovery after surgery protocols has further improved perioperative outcomes, emphasizing the importance of standardized care pathways (33, 34). Vigilance for rare but serious complications, along with specialized management approaches, continues to be essential for maintaining safety standards. The future of HIPEC in ovarian cancer is characterized by ongoing innovation across multiple domains. Novel techniques such as pressurized intraperitoneal cold atmospheric plasma and combination approaches with normothermic intraperitoneal chemotherapy or immunotherapy represent promising directions for enhancing efficacy (37, 38, 40). Drug development efforts continue to expand the available therapeutic options, while molecular profiling advances enable more personalized treatment selection (24, 39). The integration of HIPEC with targeted systemic therapies, particularly PARP inhibitors, demonstrates potential for synergistic effects that may further improve outcomes (42).

Despite these advancements, challenges remain in standardizing protocols, addressing disparities in access to care, and resolving ongoing controversies in specific clinical scenarios (45, 46). The discrepancies between international guidelines regarding HIPEC recommendations highlight the need for continued research and evidence generation (46). Future studies should focus on refining patient selection criteria, optimizing technical parameters, and evaluating HIPEC within the context of evolving systemic therapy landscapes.

In conclusion, HIPEC represents a significant advancement in the management of ovarian cancer with peritoneal dissemination. When applied to appropriately selected patients by experienced multidisciplinary teams, it offers improved survival outcomes with acceptable morbidity. As research continues to refine patient selection, technique, and integration with systemic therapies, the role of HIPEC in ovarian cancer management is likely to expand, offering hope for improved outcomes in this challenging disease.
