# Supplementary figures and images for: Hyperthermic intraperitoneal chemotherapy in ovarian cancer: a comprehensive review
Source: Front Oncol. 2026 Jan 12;15:1714997. doi: 10.3389/fonc.2025.1714997 (PMC12832346; doi:10.3389/fonc.2025.1714997)

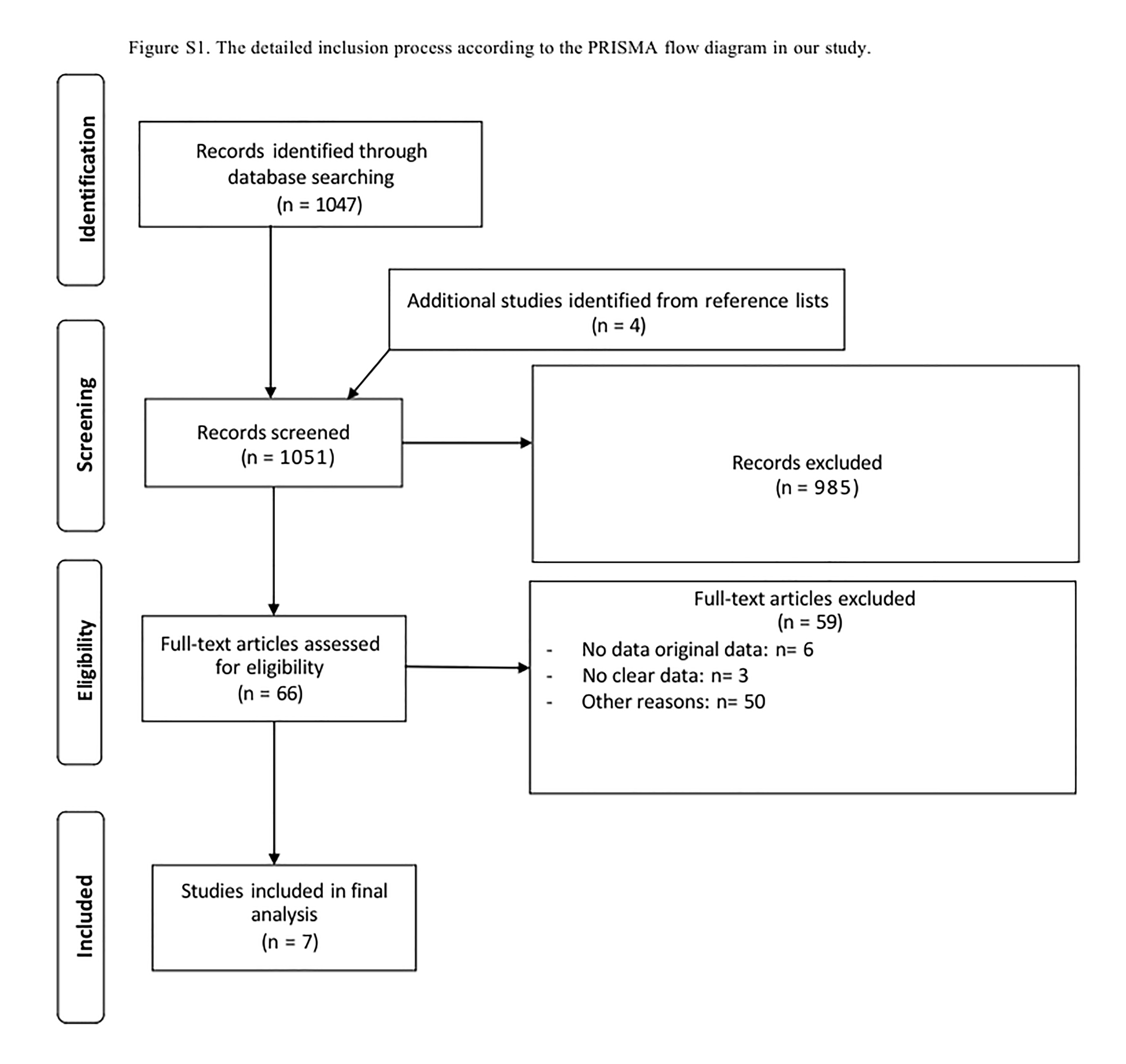

Supplement: Supplementary file 1 [file Image1.tif]
